# Objective assessment of surgical technique in rotation and nasal projection variation^☆^^☆☆^

**DOI:** 10.1016/j.bjorl.2015.11.003

**Published:** 2015-12-10

**Authors:** Marina Serrato Coelho Fagundes, Ana Tereza Moreira, Elizabeth Mila Tambara, Sérgio Bernardo Tenório, Rogério de Fraga, Rogerio Hamerschmidt

**Affiliations:** Surgical Clinic, Universidade Federal do Paraná (UFPR), Curitiba, PR, Brazil

**Keywords:** Rhinoplasty, Measures, Suture techniques, Rinoplastia, Medidas, Técnicas de sutura

## Abstract

**Introduction:**

In rhinoplasty, facial esthetic analysis is critical for proper surgical planning. Parameters such as rotation and nasal projection should be routinely evaluated. Few studies have objectively assessed changes in facial angles postoperatively.

**Objective:**

To evaluate the effectiveness of medial intercrural sutures and of rotation of the nasal tip on the increase of rotation and nasal projection in Caucasian patients undergoing primary rhinoplasty.

**Methods:**

A prospective study carried out between 2011 and 2013, with 27 patients treated with primary rhinoplasty with a basic technique by the same surgeon, with medial intercrural sutures and rotation of the nasal tip. Rotation and nasal projection were measured from photographs obtained preoperatively and after 12 months.

**Results:**

All 27 patients completed the study. The mean age was 27.1 years. There was a mean increase of 8.4° in the rotation – a statistically significant value. There was no significant change in the projection.

**Conclusion:**

The medial intercrural and nasal tip rotation sutures are effective in increasing nasal rotation in Caucasian patients undergoing rhinoplasty.

## Resumo

**Introdução:**

Na rinoplastia, a análise estética facial é essencial para um planejamento cirúrgico adequado. Parâmetros como rotação e projeção nasal devem ser rotineiramente avaliados. Há poucos estudos que avaliem objetivamente as mudanças nos ângulos faciais no pós-operatório.

**Objetivo:**

Avaliar a eficácia das suturas intercrura mediais e de rotação da ponta nasal no aumento da rotação e projeção nasal em pacientes caucasianos submetidos à rinoplastia primária.

**Método:**

Estudo prospectivo realizado entre 2011 e 2013, com 27 pacientes submetidos à rinoplastia primária técnica básica, pelo mesmo cirurgião, com suturas intercrura mediais e de rotação da ponta nasal. Mediu-se a rotação e a projeção nasal a partir de fotografias no pré-operatório e 12 meses após.

**Resultados:**

Todos os 27 pacientes completaram o estudo. A idade média foi de 27,1 anos. Houve aumento médio de 8,4° na rotação, com significância estatística. Não houve alteração significativa na projeção.

**Conclusão:**

As suturas intercrura mediais e de rotação da ponta nasal são eficazes no aumento da rotação nasal em pacientes caucasianos submetidos à rinoplastia.

## Introduction

Rhinoplasty is one of the most challenging cosmetic surgeries of the face. The surgeon must combine the correction of functional changes with his/her sense of beauty and harmony, taking into account the patient's wishes.^1^ A facial esthetic analysis is essential in this context, in which the nasal anthropometric measurements should be assessed and, from them, a surgical plan should be created so that the desired results can be achieved. Currently, the parameters used in facial esthetic surgeries are based on the study of Powell and Humphreys.^2^ These authors formulated suitable relations between face and nose, defining facial angles.

Nasal ptosis and a lack of tip projection represent a large portion of the complaints of patients, and can reach 72%.^3^, ^4^ In assessing nasal ptosis, one should observe the rotation of the nasal tip through the nasolabial angle. This angle is obtained by the angular inclination of columella at the point where it meets a line tangent to the upper lip. A nasolabial angle is deemed optimal when it falls between 90° and 105° in men and 105° and 120° in women.^5^

The projection of the nasal tip is the distance at which the nasal tip protrudes from the face. It is measured by a ratio. There are various methods of evaluating this ratio, among them the Goode method. This method is performed by measuring the line perpendicular to the tip point to the line of the facial plane divided by the nasion line to the tip point. It is recommended that this value falls in the range of 0.55–0.6.^6^

Several approaches and techniques can be used in the management of rotation and nasal tip projection changes. The literature contains descriptions of open and closed (endonasal) techniques, and the use of grafts and sutures. The endonasal technique produces the same results of the open technique, though with less damage to the support mechanisms of the nasal tip.

The nasal support mechanisms are divided into major and minor mechanisms. Major mechanisms include the fibrous ligament of the cephalic rim of the alar cartilage to the caudal rim of upper lateral cartilage; the shape, size and strength of medial and lateral crura, and the fibrous ligament from the medial crus to the caudal rim of the quadrangular cartilage. The minor support mechanisms are the cartilaginous dorsum of the septum, the membranous septum, nasal spine, the interdomal ligament, sesamoid complexes of lower lateral cartilages, ligaments between the lower lateral cartilages, and skin/soft tissues.^6^

Coupled with the knowledge of nasal support mechanisms, knowledge of their dynamics also is important. The idea that a change in rotation and projection of the nasal tip could occur due to changes in medial or lateral crura was first described in 1960, as the tripod concept.^7^ In 1969, Anderson described this theory, in which the lateral crus of each lower lateral cartilage and the two medial crura form a tripod. Therefore, two legs of this tripod are formed by the lateral crura, and the third leg is formed by the medial crura and columella. In this case, by changing the length and position of each leg, the positioning of the nasal tip will be impacted.^5^, ^8^ Adamson extended this theory and proposed a M-arc model, in which the medial and lateral crura are considered as part of an arc of defined length, also taking into account the distance between the domus.^7^, ^8^ These theories are complementary, and are essential when planning a nasal surgery.

The use of nasal sutures is becoming an increasingly popular option, is easy to learn and has a low risk of complications. In the literature, studies that objectively evaluate the changes in facial angles postoperatively are scarce.^5^ In our study, preoperatively, and 12 months after the procedure, we use an endonasal approach to objectively place and later evaluate the medial intercrural suture associated with the cephalic rotation suture, for increasing and maintaining the projection and nasal tip rotation. These sutures are modifications of previously described techniques.^9^

The medial intercrural suture was originally described as a technique to increase both the projection and the rotation of the nose tip. The nasal tip rotation suture has also been described, with the goal of increasing head rotation and promoting a slight retraction of columella.^9^

This study aims to evaluate measures of rotation and nasal tip projection with the use of medial intercrural and nasal tip rotation sutures at the twelfth postoperative month following primary endonasal rhinoplasty.

## Methods

This study was approved by the ethics committee of the institution (003/2012-06).

Data collection was performed prospectively from January 2011 to December 2013 by the researcher.

Twenty-seven patients were selected in a study of a contemporary longitudinal cohort. Patients included in the study had noses with predominantly Caucasian features complaining of little rotation and/or nasal projection. Exclusion criteria: patients who had undergone previous rhinoplasty, and those aged under 18. Prior to surgery, all patients signed a free and informed consent form approved by the ethics committee. No patient was lost to follow-up.

The selected patients underwent a basic technique rhinoplasty (Table 1) performed by the same surgeon, with application of medial intercrural and nasal tip rotation sutures. The septocolumellar suture, to close the access incision, was carried out at the same level.

**Table 1:** Basic technique for rhinoplasty. Steps of basic technique for rhinoplasty.

Basic technique for rhinoplasty
Septocolumellar and intercartilaginous incision
Resection of membranous septum
Detachment of the nasal dorsum and periosteum
Upper lateral cartilages splitting
Hump removal
Lateral fracture
Dissection of columellar pocket
Sutures

**Figure 1:**
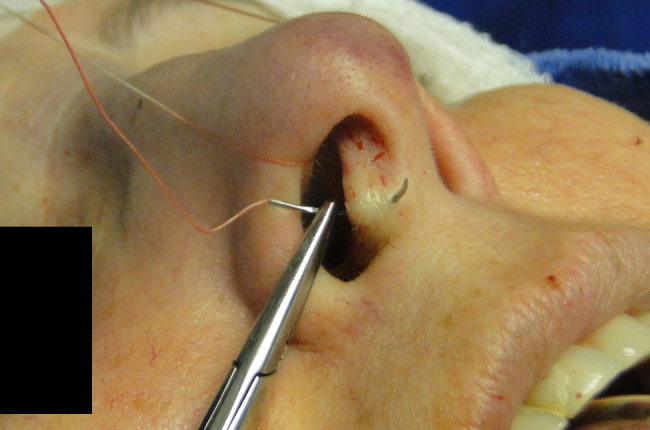
Intercrural medial suture. Step 1 – passage with a 5 mm curved needle above the base of columellar pocket to the external aspect.

**Figure 2:**
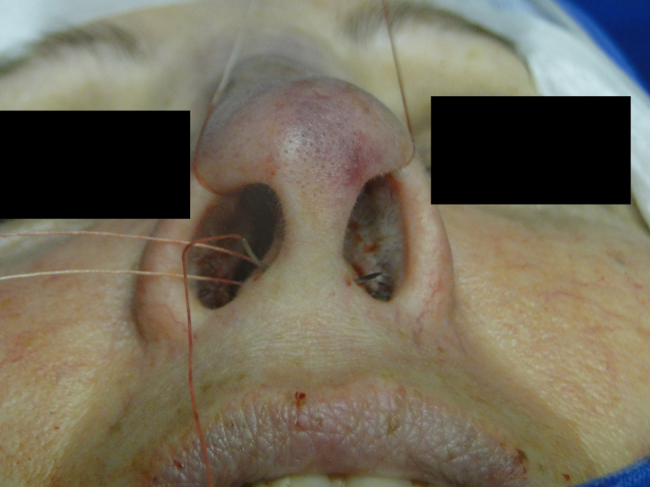
Intercrural medial suture. Step 2 – using the same exit orifice, the needle traverses the columella and exits at the same level on the opposite side.

**Figure 3:**
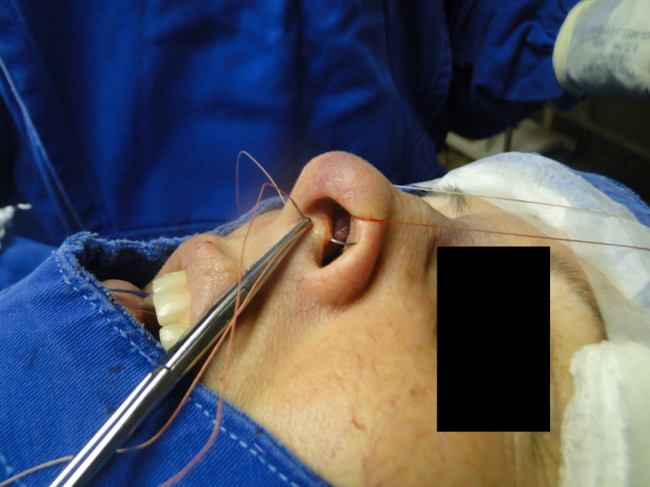
Intercrural medial suture. Step 3 – through the same orifice, the needle returns to the interior of the pocket.

**Figure 4:**
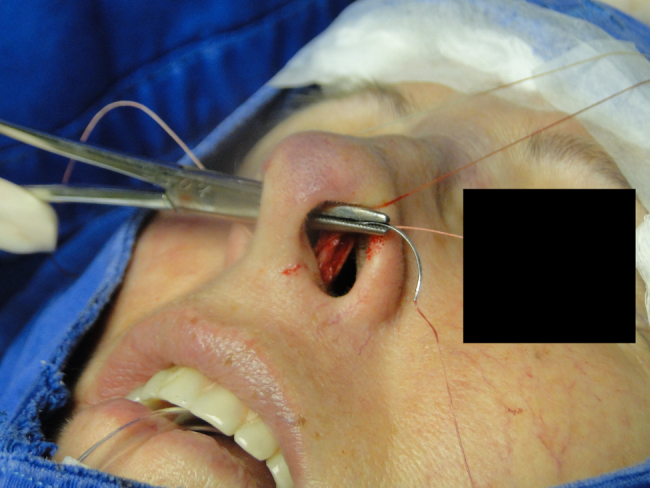
Intercrural medial suture. Step 4 – passage of the needle to the side where the suture began.

**Figure 5:**
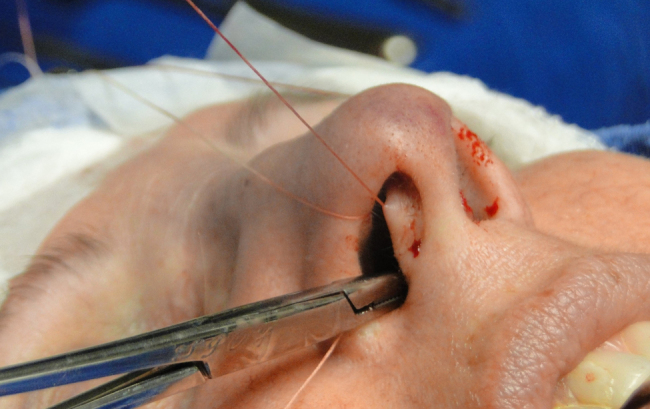
Intercrural medial suture. Step 5 – tying three fixation knots.

**Figure 6:**
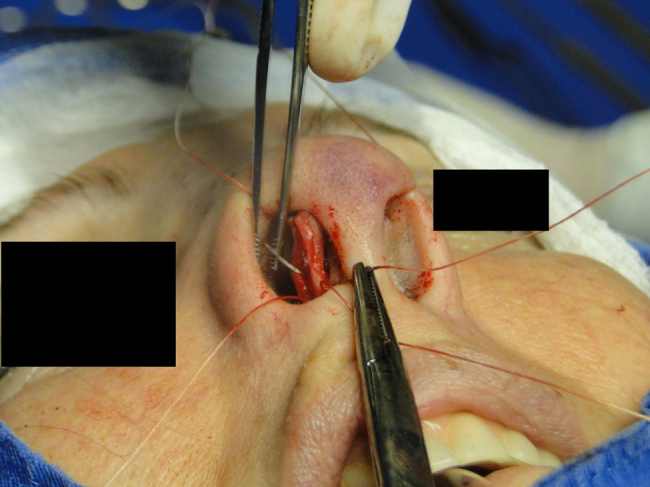
Intercrural medial suture. Step 6 – passing the point through septal cartilage, 5 mm posterior to the caudal rim.

The medial intercrural suture is performed with a clear Nylon 4.0 filament after columellar pocket detachment, as shown in the figures below. The 5 mm curved needle is passed above the inside of the base of the columellar pocket toward the external region (Fig. 1), where, by the same exit orifice, the needle passes through the columella and exits at the same level on the opposite side (Fig. 2). Using the same orifice, the needle returns to the inside of the pocket, where three approximation knots are tied (Figure 3, Figure 4, Figure 5). Then, this stitch is passed through septal cartilage, in a point 5 mm posterior to its caudal rim, ending with three knots (Fig. 6). The suture is buried in the mucosa, without involvement of this structure. In the original description, the approximation knots are not performed.

**Figure 7:**
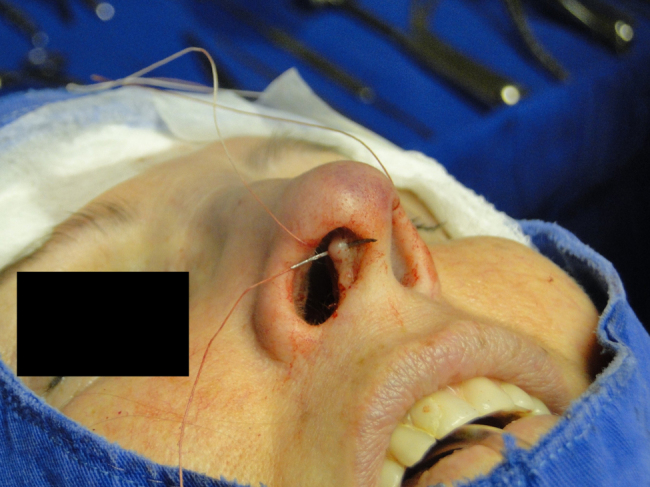
Nasal tip rotation suture. Step 1–5 mm below the apex of the columella, the needle is passed from the pocket to the external aspect.

**Figure 8:**
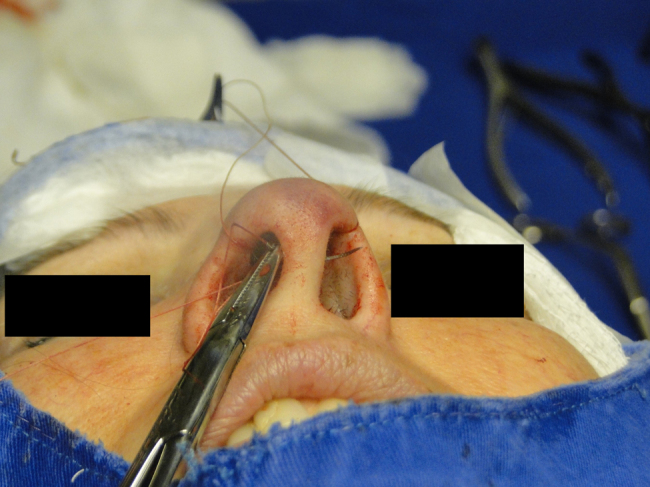
Nasal tip rotation suture. Step 2 – the needle returns through the same orifice to the opposite side, at the same level.

**Figure 9:**
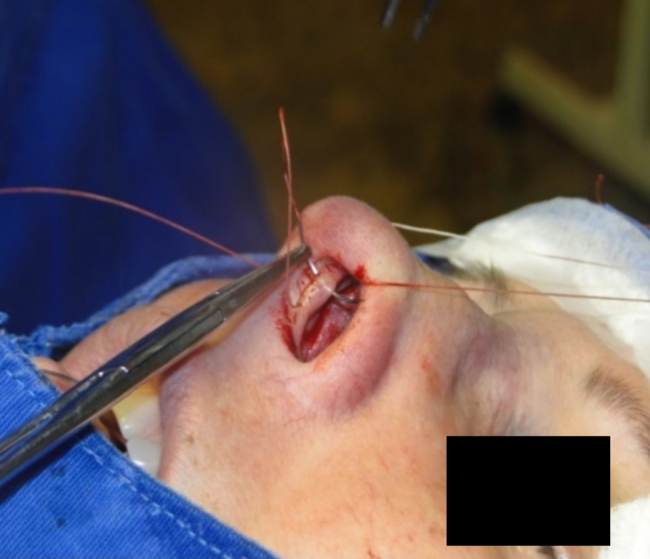
Nasal tip rotation suture. Step 3 – through the same orifice, the needle returns to the pocket.

**Figure 10:**
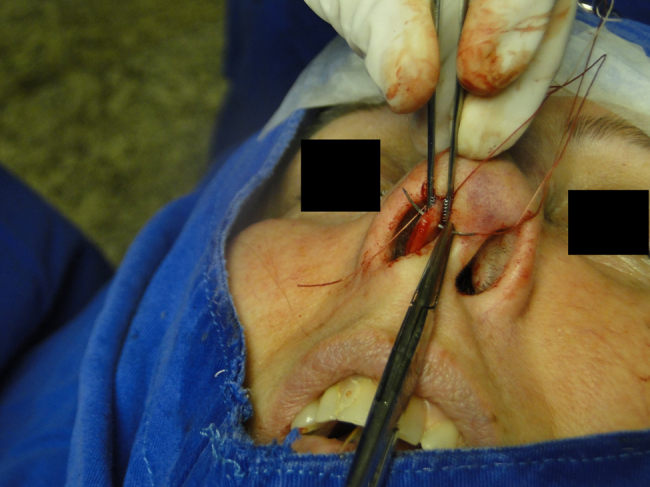
Nasal tip rotation suture. Step 4 – the needle is trespassed 5 mm behind and below the nasoseptal angle.

In the cephalic rotation suture, which is applied at a distance of 5 mm below the columellar apex, the needle is passed from the pocket to the external region (Fig. 7), returning through the same hole to the opposite side, at the same level (Fig. 8), where, through the same hole, the needle returns to the pocket (Fig. 9); in sequence, the suture is trespassed at a point 5 mm behind and below the nasoseptal angle, ending with three knots (Fig. 10). The suture is buried in the mucosa, also without involvement of this structure.

**Figure 11:**
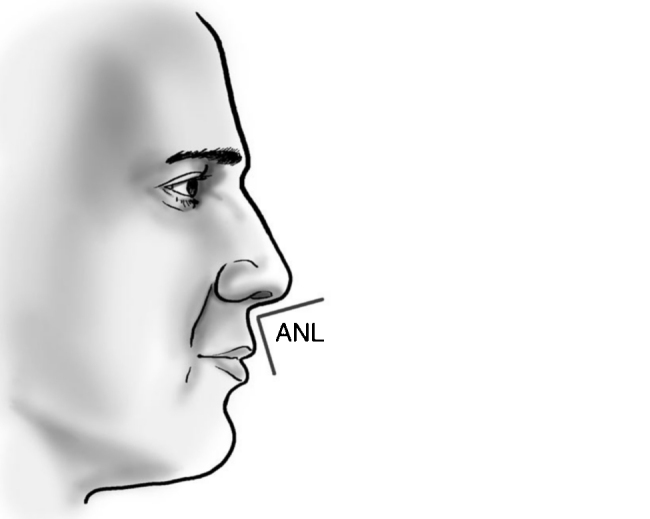
Nasolabial angle. Analysis of nasal rotation.

**Figure 12:**
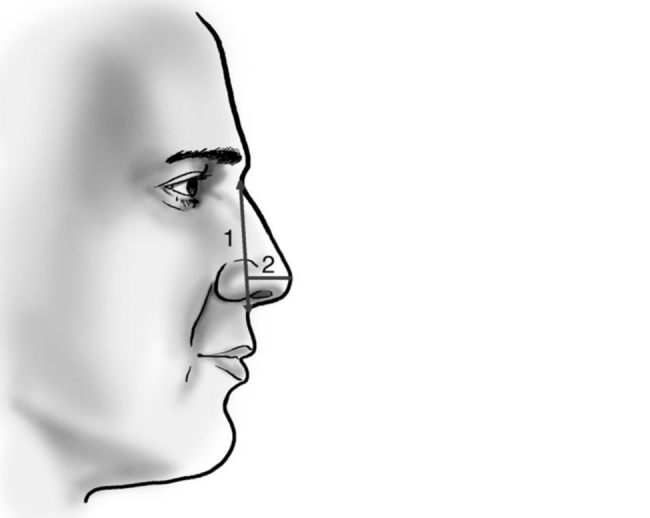
Nasal projection. Analysis of nasal projection.

**Figure 13:**
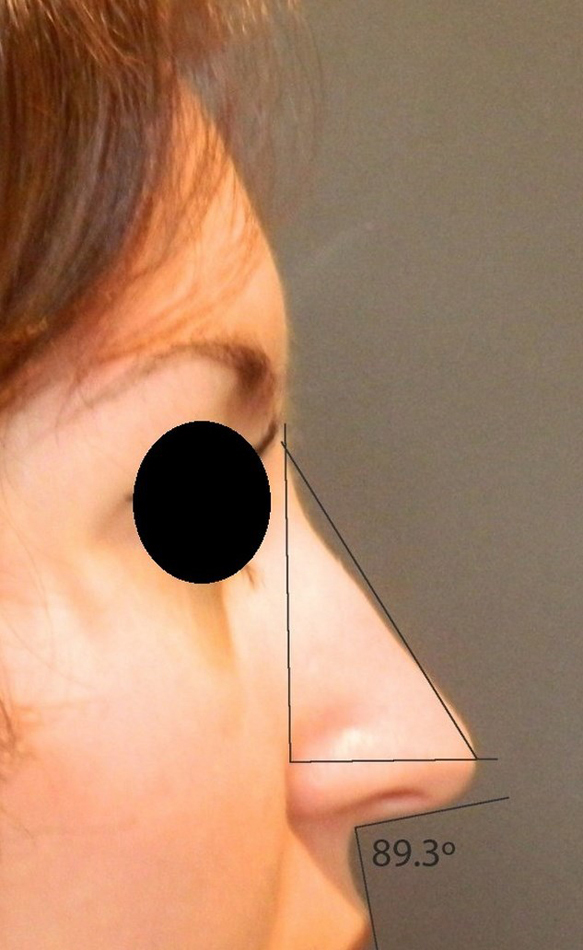
Preoperative. Nasal measures in preoperative period.

**Figure 14:**
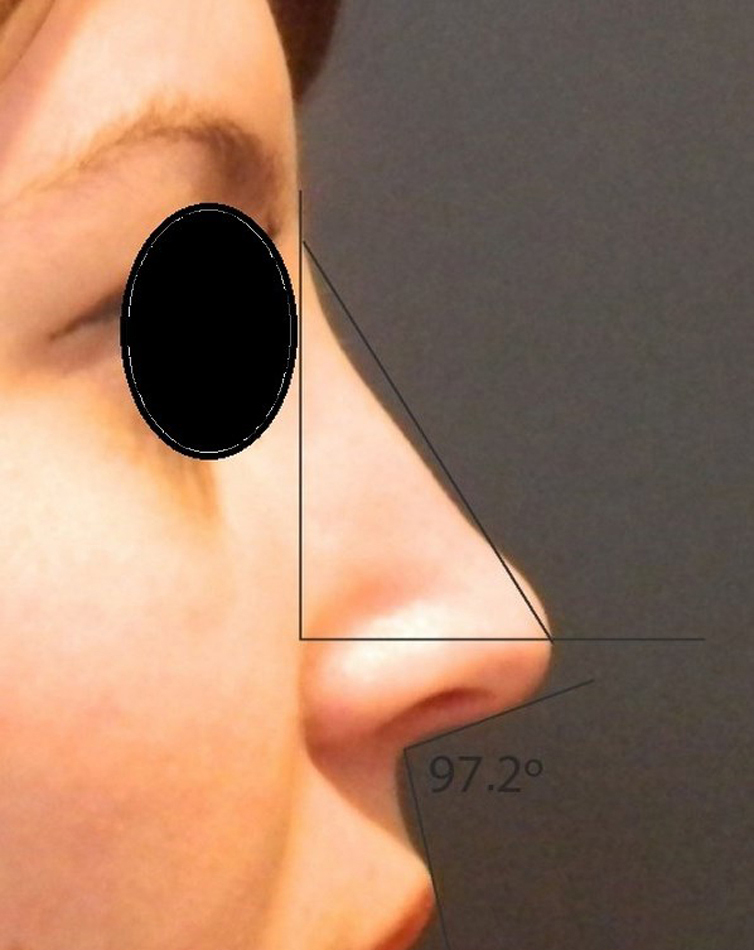
Postoperative. Nasal measures in late postoperative period.

**Figure 15:**
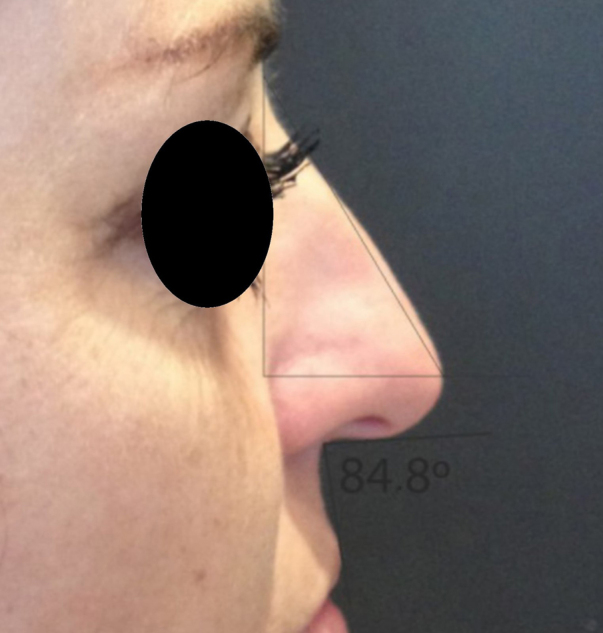
Preoperative. Nasal measures in preoperative period.

**Figure 16:**
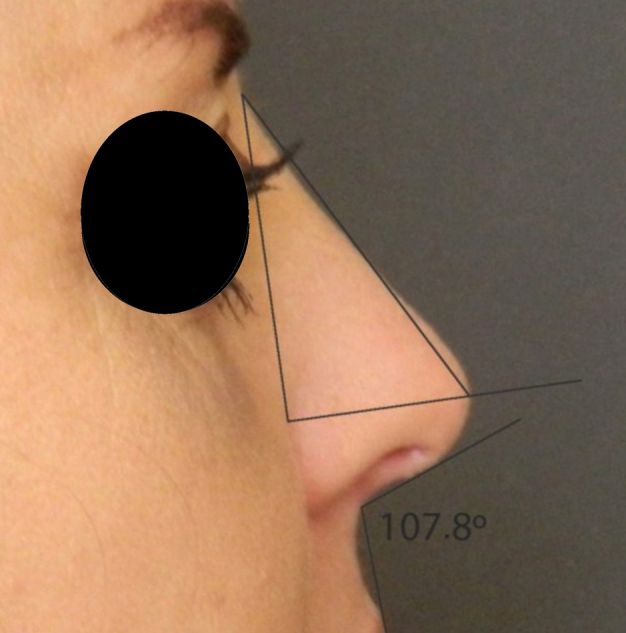
Postoperative. Nasal measures in postoperative period.

**Figure 17:**
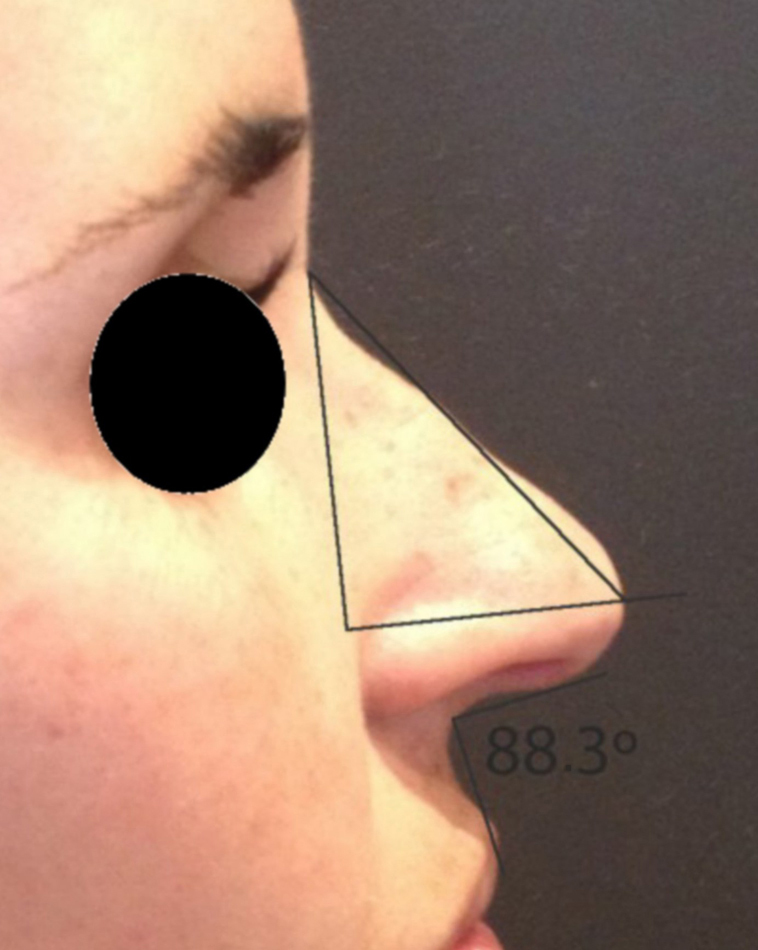
Preoperative. Nasal measures in preoperative period.

**Figure 18:**
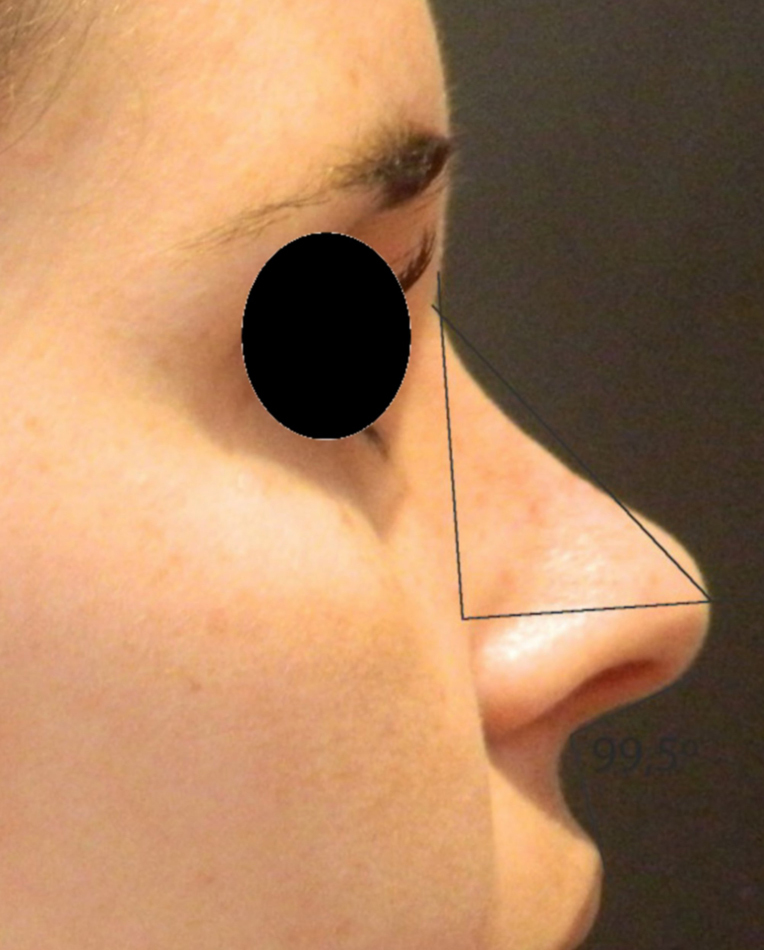
Postoperative. Nasal measures in postoperative period.

Rotation (Fig. 11) and nasal tip projection (Fig. 12) measures were obtained from photographs preoperatively and after 12 postoperative months. All pictures were taken by the same researcher using a dark background in a right lateral view, with the use of a Nikon Collpix 120 camera without tripod and accessory lighting. These photos were analyzed using Photoshop CS3's metric tool with calculation of coefficients through proportion relationships; thus, there was no distortion of the variables analyzed. For the nasal rotation measurement, two tangents to the columella and to the upper lip were drawn, and the angle of contact between these two lines determined the amount of rotation. To measure nasal projection, we used the Goode method, by measuring the line perpendicular from the tip point to the facial plane line, divided by the length of the line from the nasion to the tip point (Figure 13, Figure 14, Figure 15, Figure 16, Figure 17, Figure 18).

A statistical analysis was carried out. For a description of quantitative variables, mean, median, minimum value, maximum value and standard deviation statistics were taken into account. For evaluation of variables’ normality condition, Shapiro–Wilk test was considered. For assessment of whether or not a difference between time points of assessment (pre and post) existed, in relation to the variables of interest, the Student's *t*-test for paired samples was applied. *p*-Values <0.05 were considered statistically significant.

## Results

The sample used in this study consisted of 27 people, including 9 males (33.3%) and 18 females (67.7%).

We tested the null hypothesis of equal mean angles in both assessment time points (pre and post) versus the alternative hypothesis of different mean angles. Nasal rotation showed a statistically significant increase (Table 2).

**Table 2:** Descriptive statistical analysis of nasal rotation.

Timing	Nr. of cases	Mean	Median	Minimum	Maximum	Standard deviation	*p*-Value^a^
Pre	27	96.8	96.6	77.4	111.6	9.2	
Post	27	105.2	106.6	85.1	122.1	8.3	
Post-pre	27	8.4	8.7	−7.4	23.0	6.8	<0.001

a Student's *t*-test for paired samples; *p* < 0.05.

We tested the null hypothesis of equal mean projections in both assessment time points (pre and post) versus the alternative hypothesis of different mean projections. There were no statistically significant changes with respect to nasal projection (Table 3).

**Table 3:** Descriptive statistical analysis of nasal projection.

Timing	Nr. of cases	Mean	Median	Minimum	Maximum	Standard deviation	*p*-Value^a^
Pre	27	0.69	0.69	0.49	0.85	0.09	
Post	27	0.67	0.66	0.55	0.85	0.07	
Post-pre	27	−0.02	−0.02	−0.20	0.16	0.08	0.184

a Student's *t*-test for paired samples; *p* < 0.05.

## Discussion

In recent years, techniques used in rhinoplasty have changed from more invasive procedures with removal of structures, to more conservative procedures focused on repositioning and structuring of existing tissues. This occurred mainly because of the development of late complications resulting from nasal healing, that sometimes caused changes in nasal form and function.^10^, ^11^

The incorporation of the measures planned in the immediate pre-operative period is a major challenge in rhinoplasty. When planning the optimal approach during surgery the surgeon should anticipate the healing forces and take into account the support mechanisms of the nasal tip.^1^

The rhinoplasty should focus on proper levels of stability, symmetry, rotation and projection that will resist nose healing forces, with maintenance of satisfactory long-term results. An inappropriate resection of cartilage and the use of grafts and sutures can lead to nasal tip deformities, including ptosis, alar collapse, retraction, and pinching.^8^

Several techniques are described for the treatment of nasal tip ptosis, including surgical procedures and minimally invasive actions, such as the application of botulinum toxin. The surgical access can be carried out through an endonasal approach, or an external technique.^3^ Open rhinoplasty has risks inherent to the procedure, such as greater damage to nasal support mechanisms and the frequent need for multiple grafts, which carry a risk of migration and exposure of the graft, especially in thin-skinned patients.^10^

The literature describes numerous maneuvers to correct noses with little rotation and projection, including cephalic resection of upper lateral cartilage, sliding of lateral crus, tongue-in-groove, grafts, and membranous septum resection.^8^

Zuliani^3^ uses the open rhinoplasty associated with lower to upper lateral cartilage suspension (LUCS) in cases of nasal ptosis; 24 patients changed their nasolabial angle from 83.4° preoperatively to 102.3° one year after surgery. When compared to the use of sutures presented in this study, the technique reported by Zuliani has shown a more significant increase in nasal rotation, but with a more aggressive technique that required an external access.

In a retrospective study of 62 patients undergoing external rhinoplasty, Ingels and Orhan^5^ concluded that six months after surgery, the use of the columellar post increased the rotation and projection of the nasal tip, an effect which is accentuated by removing the cephalic portion of the upper lateral cartilages. This increase was not measured, and both the sample and the technique were not standardized.

The tongue-in-groove technique gives good results in patients undergoing rhinoplasty, with little rotation of the nasal tip, but with a proper projection.^8^ The results were evaluated subjectively, preventing comparisons and future standardizations.

Patrocínio^12^ evaluated 20 patients who underwent rhinoplasty with the use of a “lateral crura steal” technique to increase nasal rotation in the late postoperative period, with a mean increase of 20° in the late postoperative period, a statistically significant outcome.

The columellar post is a rectangular autologous graft that is fixed between the medial crura, with the aim to support and maintain the projection and rotation of the nasal tip. This structure is routinely used when performing an open technique, due to the inherent injury of the support mechanisms, but may also be used in endonasal approaches.^13^ Toriumi^11^ described the importance of suturing the caudal septum in the medial crura (tongue-and-groove) to stabilize the nasal base, in association with the use of the columellar post. Combined with a higher degree of injury of structures for achieving access, there are still complications resulting from the use of the graft. The complications encountered with the use of grafts include a bad position, displacement, induration, resorption, visible irregularities, extrusion, infection, atrophy, and soft tissue deformities.^14^, ^15^

The use of the horizontal columellar post proposed by Margulis^4^ was effective in stabilizing the nasal tip in patients with severe ptosis at the tip. The use of shell-type grafts increases nasal projection; however, this option should be avoided in patients with thin skin, due to the risk of exposure and extrusion.^11^ This type of graft should be camouflaged, especially in patients with thin skin. The use of sutures is very advantageous for these patients, because there is no marking on the overlying skin.

In 2011, Cingi^14^ described the triple suture of cartilage, a modification of the tongue-in-groove technique, with the use of figure-of-eight sutures – a technique that did not exhibit complications and had very good subjective results. This technique improves the rotation of the nasal tip, presents a gradual adjustment and allows the correction of an excessive columella. The authors did not standardize their measures, complicating comparisons with other techniques.

For endonasal correction of hypoprojected tips, various techniques have been described by Pastorek^16^ including interdomal suture, use of a columellar post, pre-maxillary grafts and an extended columellar graft, all with satisfactory results.

The original description of sutures used in this study was made by Guyron^17^ in 1998; this author described the suture at the base of medial crura and soft tissue removal between the crura, as well as the use of U-sutures for bringing the feet together, assuming that the correction of hypoprojection should involve medial crura, according to the tripod model.^6^ In this study, initially three approximation knots were applied to achieve a better columellar contour, with a final esthetic effect similar to that achieved by the technique of columellar post placement, in addition to an association with the suture of rotation of the nasal tip. This technique differs from the usual septocolumellar suture, since it allows greater medial crura stabilization, combined with the possibility of greater unevenness of height between stitches, without affecting the overlying mucosa, since all the sutures and knots remain buried.

In an objective study of the endonasal rhinoplasty conducted by Pasinato,^2^ the author measured the angles, both in preoperative and in early postoperative periods, and reported an increase of 8.6° for the nasolabial angle after the procedure. There was neither comparison with the technique performed, nor standardization among patients.

The nasal techniques using sutures to increase rotation and nasal tip projection have enjoyed an increase in popularity, appearing as an option in both open and endonasal rhinoplasty.^3^ These techniques have predictable and controlled results, and they meet the most current principles of rhinoplasty – conservation of structures with non-destructive techniques.^3^, ^10^ Many surgeons agree that reversible, non-destructive, and structure-repositioning techniques should be used in preference to more aggressive techniques and of an indiscriminate use of grafts.^7^ Remodeling and positioning of structures can be accomplished through sutures, without an unnecessary sacrifice of structures.^7^, ^15^

The medial intercrural suture can both increase or decrease nosal tip projection, depending on where the suture is anchored to the nasal septum.^9^ Patients with a nasal base lacking adequate structure are more likely to exhibit hypoprojected and hyporotated noses. Stabilization of the nasal base may assist in increasing nasal projection and rotation, and in maintaining an increased nasolabial angle. Nasal base stabilization is an essential step in the long-term maintenance of nasal tip position.^11^ In this study, a distance of 5 mm posterior to the caudal septum was standardized, although the height was not, and the anchoring point was applied at the same level of the medial crural suture, which may have resulted in the absence of significant changes in nasal projection. If the suture is anchored in the nasal spine area, there will be a potential for reduction of projection and also of nasal rotation. In this study, there is also the potential for a lack of change in nasal projection because of a beta error from an insufficient sample size. We did not observe the slight retraction initially described by Behmand, Ghavani, and Guyuron,^9^ probably because the first suture for the approximation of the medial crura does not allow the septum to exceed this limit during knot anchoring in the sequence of the stitch – a procedure not described in the original technique. A decrease of lobule and columella also occurs.

The nasal tip rotation suture, as the name implies, increases the cephalic rotation of the nasal tip and can also result in columellar narrowing. A slight flattening of the columella can also occur.^9^

The effects of the sutures are influenced by the degree of their tightening, the thickness of the cartilage, the amount of subcutaneous tissue and the thickness of the skin.^9^ There are numerous techniques described to increase the rotation and projection of the nasal tip; however, there are few objective reports after surgery, especially long term, to allow one to quantify the effectiveness of these techniques.^5^ This hinders future comparative studies and the selection of the optimal technique for each patient, according to the result to be achieved.

Modifications occurring in terms of tip rotation may be real, in which the nose tip position is changed, or illusory, through changes in the dorsum, columella, and in the contour of the tip itself.^11^

In addition to the cartilaginous–osseous structure, the appearance of nasal tip also depends on subcutaneous tissue. In the case of weak cartilage, with thick skin and abundant subcutaneous tissue, the results are usually poor. It should be noted that the lower lateral cartilages are the main supporting structures of the nasal tip. Therefore, any excess, deficiency or change in these cartilages will directly affect the overlying shape of the skin.^9^

The long-term results are more predictable and reliable with the use of precisely positioned sutures, and with an understanding of their interaction with the nasal tip whether they are used alone or in combination.^15^ These sutures should be tied at the completion of surgery, so that there is not excessive handling or excessive use of the speculum to risk breaking them. This technique presents little risk of complications, considering that during the intraoperative period their results can already be observed, and when necessary, these sutures can be undone and applied again, until an optimal esthetic result is achieved. It is worth noting that the tightening of the knots is a critical step, and for best results it should be done gradually. In this study, we observed one case of columella infection, which responded promptly to oral antibiotics.

In this study, there was no combined use of these sutures with other stitches, because of its main objective; however, this can be done and has been widely used in recent years, with quite safe and predictable results.^9^ It is worth noting that this is a non-comparative study, and that future studies can be performed to compare this technique with other procedures, perhaps facilitating and more precisely tailoring the surgical technique to be used, depending on the specific gain for the patient and avoiding the use of more aggressive techniques (when there is no need), and of less effective techniques in noses requiring more significant changes.

The sutures are considered indispensable for a refinement of the nasal tip surgery.^18^

## Conclusion

This study denomstrated that medial intercrural and nasal tip rotation sutures are effective in increasing the nasolabial angle in Caucasian patients undergoing primary endonasal rhinoplasty. The sutures did not have a significant effect on nasal projection.

## Conflicts of interests

The authors declare no conflicts of interest.
